# Low-Intensity Physical Exercise Improves Pain Catastrophizing and Other Psychological and Physical Aspects in Women with Fibromyalgia: A Randomized Controlled Trial

**DOI:** 10.3390/ijerph17103634

**Published:** 2020-05-21

**Authors:** Ruth Izquierdo-Alventosa, Marta Inglés, Sara Cortés-Amador, Lucia Gimeno-Mallench, Javier Chirivella-Garrido, Juri Kropotov, Pilar Serra-Añó

**Affiliations:** 1 Unidad de Biomecánica Clínica (UBIC Research Group), Department of Physiotherapy, Faculty of Physiotherapy, Universitat de València, 46010 València, Spain; ruthizquierdo91@gmail.com (R.I.-A.); sara.cortes@uv.es (S.C.-A.); pilar.serra@uv.es (P.S.-A.); 2 Freshage Research Group, Department of Physiotherapy, Faculty of Physiotherapy, Universitat de València, Centro de Investigación Biomédica en Red Fragilidad y Envejecimiento Saludable (CIBERFES-ISCIII), Fundación Investigación del Hospital Clínico Universitario de Valencia (INCLIVA), 46010 València, Spain; 3 Freshage Research Group, Department of Physiology, Faculty of Medicine, Universitat de València, Centro de Investigación Biomédica en Red Fragilidad y Envejecimiento Saludable (CIBERFES-ISCIII), Fundación Investigación del Hospital Clínico Universitario de Valencia (INCLIVA), 46010 València, Spain; lucia.gimeno@uv.es; 4 Fundación Fivan, Centro de Neurorehabilitación, 46005 Valencia, Spain; xiri@mac.com; 5 N.P. Bechtereva Institute of Human Brain, Russian Academy of Science, 197022 St. Petersburg, Russia; yurykropotov@yahoo.com

**Keywords:** fibromyalgia, pain catastrophizing, physical exercise

## Abstract

Fibromyalgia (FM) is a chronic syndrome characterized by widespread pain and other physical and psychological features. In this study, we aimed to analyze the effect of a low-intensity physical exercise (PE) program, combining endurance training and coordination, on psychological aspects (i.e., pain catastrophizing, anxiety, depression, stress), pain perception (i.e., pain acceptance, pressure pain threshold (PPT), and quality of life and physical conditioning (i.e., self-perceived functional capacity, endurance and functional capacity, power and velocity) in women with FM. For this purpose, a randomized controlled trial was carried out. Thirty-two women with FM were randomly allocated to a PE group (PEG, n = 16), performing an eight-week low-intensity PE program and a control group (CG, n = 16). Pain catastrophizing, anxiety, depression, stress, pain acceptance, PPT, quality of life, self-perceived functional capacity, endurance and functional capacity, power, and velocity were assessed before and after the intervention. We observed a significant improvement in all studied variables in the PEG after the intervention (*p* < 0.05). In contrast, the CG showed no improvements in any variable, which further displayed poorer values for PPT (*p* < 0.05). In conclusion, a low-intensity combined PE program, including endurance training and coordination, improves psychological variables, pain perception, quality of life, and physical conditioning in women with FM.

## 1. Introduction

Fibromyalgia (FM) is a chronic condition characterized by widespread pain associated with other physical symptoms, such as fatigue or decreased physical capacity, and psychological alterations [1]. One of the psychological alterations that has been associated with FM is pain catastrophizing, a specific psychosocial construct of pain, which includes cognitive and emotional processing, sense of helplessness, pessimism, and rumination about pain-related symptoms [2]. Pain catastrophizing has been associated with pain severity and disability [3], which is being considered a risk factor for pain chronification [4]. Furthermore, this construct of pain has been shown to decrease pain acceptance, which, in turn, may aggravate the symptomatology of FM [5]. Pain acceptance is lower in FM patients [6], which has been linked to a higher degree of disability [7] and a lower quality of life [8].

In addition to pain catastrophizing, other psychological alterations that can aggravate the symptomatology of FM are anxiety and depression. These alterations, together with high levels of stress, have been proposed as precipitating and/or perpetuating factors of this condition [9] and are inversely related to quality of life among FM patients [10]. In this regard, it has been suggested that the higher the level of pain catastrophizing, anxiety, and depression in FM individuals, the greater their sensitivity to non-painful stimuli and difficulty in coping with the painful process [11].

Interestingly, pain catastrophizing, has also been inversely related to muscular endurance [12]. This tendency has proven to have a negative impact on neuromuscular, cardiovascular, immune, and neuroendocrine systems [13]. In turn, such an impact causes an alteration of functional capacity [4], which can be assessed both objectively and subjectively. An objective decline in physical conditioning has a detrimental effect on the ability to perform activities of daily life, but also the subjectively altered perception of functional capacity can lead to actual physical inactivity and a progressive deconditioning [14]. Physical deconditioning may negatively impact the individual’s quality of life [15] and his/her professional performance, which leads to absenteeism [16]. 

Since a direct relationship between health care costs and severity of FM symptoms has been documented [17], implementing an effective therapeutic approach remains a paramount challenge for the medical community. Current FM management is usually based on pharmacological treatment, which, despite being equally effective as a non-pharmacological therapy, has greater side effects and lower acceptance by FM patients [18]. One of the most promising and cost-effective non-pharmacological approaches is physical exercise (PE). Thus, a number of protocols have been proposed, such as aerobic [19,20,21,22,23] resistance [19,22,24,25,26,27,28], flexibility [24,26,28], combined [20,29,30,31,32,33,34], or other modalities [23,35,36], which have achieved improvements mainly in quality of life, pain, fitness, and depression. Overall, it has been suggested that a protocol including endurance and coordination would be the treatment of choice [37] with progressive workloads adapted to the individual’s condition to promote adherence [38].

In this regard, to the best of our knowledge, no previous study has been carried out to analyze the impact of a low-intensity exercise program, combining endurance training, i.e., aerobic and resistance exercises aimed at improving endurance and coordination, and adapted to the symptomatology of patients (i.e., individualized and progressive) on pain catastrophizing and other psychological variables such as pain acceptance or self-perceived functional capacity in women with FM. Given the previously mentioned deleterious effects of the negative cognitions on FM symptoms, we hypothesized that a low intensity PE program would improve catastrophism in women with FM, which results in an improvement in other related psychological and physical variables. Thus, the aim of this study was to determine the effects of a low-intensity PE program, combining endurance training and coordination, on pain catastrophism in women with FM. Furthermore, we aimed to assess the effects of the proposed protocol on other psychological aspects (i.e., anxiety, depression, and stress), pain perception (i.e., pain acceptance and pressure pain threshold), quality of life, and physical conditioning (i.e., self-perceived functional capacity, endurance and functional capacity, power, and velocity) in women with FM.

## 2. Materials and Methods

### 2.1. Participants

Thirty-two women diagnosed with FM were recruited from several Fibromyalgia Associations from February to May 2019 to participate in this study. Inclusion criteria for the participants were: (i) women between 30–70 years old, an age range in which FM becomes more prevalent [39], diagnoses according to the 2016 American College of Rheumatology criteria for FM [40], and having received pharmacological treatment for more than three months with no clinical improvement. Exclusion criteria were: (i) pregnancy or breast-feeding, (ii) any known advanced-stage pathology associated with the locomotor system that contraindicates physical activity (arthritis, osteoarthritis, uric acid), (iii) epilepsy, (iv) intake of drugs that reduce the seizure threshold, (v) history of intense headaches, (vi) neurological disorder, (vii) peripheral neuropathy, (viii) known serious cardiovascular disease (i.e., endocranial hypertension, uncontrolled arterial hypertension, heart failure, cardiac pacemaker), (ix) pneumothorax, (x) neoplasia, (xi) surgery in the last four months, (xii) diagnosis of alcohol addiction, and (xiii) use of psychoactive drugs or narcotics. Moreover, patients should not have been enrolled in any PE program in the two months before the study began.

#### 2.1.1. Study Design

A randomized controlled trial was performed (NCT03801109). The participants were randomly allocated to two different groups using the simple randomization method with the Random Allocation Software [41] by an external assistant who was blinded to the study objectives: physical exercise group (PEG) (n = 16) and control group (CG) (n = 16). To analyze the effect of the interventions, two assessments were performed: one at baseline (T0) and another following the intervention (T1). The physical therapist performing the assessments was unaware of the group the patients had been assigned to. To reduce bias, participants were instructed not to tell the assessor about the treatment they received.

All enrolled participants provided informed written consent prior to entering the study. All procedures were conducted in accordance with the principles of the World Medical Association’s Declaration of Helsinki and the protocols were approved by the Ethical Committee of the *Universitat de València*.

#### 2.1.2. Sample Size Calculation

Sample size was calculated by accounting for two study groups measured twice and with reference to a previous study conducted by Koele et al. [42] in which pain catastrophizing was measured. Accordingly, an effect size of d = 0.72 was expected. Furthermore, a type I error of 5% and a type II error of 20% were set. This calculation rendered 14 volunteers per group. Ultimately, 32 women were included to prevent loss of power derived from potential dropouts. G-Power^®^ version 3.1 was used for sample size estimation (Institute for Experimental Psychology, University of Düsseldorf, Düsseldorf, Germany).

#### 2.1.3. Intervention Procedures

As reported, the participants were allocated to two groups (i.e., PEG and CG) whose interventions are explained below. During each session, potential discomfort or adverse effects, such as severe muscle pain (i.e., ≥7.5) [43] and/or excessive fatigue (i.e., ≥5) [44], were recorded using a 10-point Visual Analogue Scale and Borg Scale of Perceived Exertion, respectively.

#### 2.1.4. Low-Intensity Physical Exercise

Participants of this group were enrolled in a low-intensity PE program combining endurance training (i.e., aerobic and low-load resistance exercises aimed at improving endurance) and coordination, supervised by a physical therapist with expertise in therapeutic exercise. All training sessions were carried out at the same time of day and in the same room. The administered protocol included 16 sessions, which were performed twice a week (60 min each) for eight weeks [29]. The sessions were divided into two stages with the first (i.e., sessions 1 to 4) being devoted to the participants’ adjustment and familiarization with the exercise, and the second (i.e., sessions 5 to 16) aimed at personalized strength and coordination training. In this regard, training intensity was adjusted by controlling the individual’s self-perceived exertion using the Borg CR-10 scale [45] as explained below.

Each session was divided into three parts: warm-up, training, and cool-down. (i) Warm-up consists of walking at a slow pace and moving the main joint structures (neck, shoulders, elbows, wrists, hips, knees, and ankles) within the patient’s range of motion. (ii) Training is explained below. (iii) Cool down consists of walking at a slow pace, overall trunk stretching, and breathing deeply, while lying on the floor.

Training in the first stage (sessions 1 to 4) consisted of walking at a comfortable speed for 15 min, performing a 10-exercise circuit for 25 min, and cooling down for 20 min. Exercises were conducted using 1-kg dumbbells and weights at a velocity determined by a metronome set at 60 beats per minute. To ensure a weak or very weak perceived effort (i.e., 1–2 categories on the CR-10 Borg) [44,45], the perceived exertion was registered after each session and the work load was individually adjusted for the next session.

In the second stage (5th to 16th session), after a 10-min warm-up, the participants had to perform as many repetitions as possible in 1 min of the exercises of the 10-exercise circuit for 40 min, reporting, in this case, a perceived effort of 3–4 on the Borg scale, to ensure a moderate effort [44,45]. After this, they cooled down for 10 min.

Table 1 shows the 10-exercise circuit for both stages 1 and 2. The work load varied depending on the participant since they were allowed to adapt the exercise according to their self-perceived pain or exertion each day [1]. However, the number of repetitions always ranged between 15 and 25 according to PE recommendations proposed by the 2014 Guide for the prescription of physical exercise of The American College of Sports Medicine for improving muscle endurance [38]. The combined aerobic and resistance training exercises aimed to work on endurance and coordination. Aerobic exercises included walking and moving the main joint structures, as explained previously. Low-load resistance training was oriented to the strengthening of the upper and lower limbs using dumbbells/weights with loads ranging between 0.5 and 2 kg for the upper limbs, and between 1 and 3 kg for the lower limbs based on the Borg scale scoring. A soft elastic band was also used for limb and trunk training and coordination exercises, as described in Table 1. Coordination exercises included standing calf raises, sitting down and standing up from a chair, stepping up and down, and throwing a ball into the air.

#### 2.1.5. Control Group

The participants assigned to this group received no intervention and were asked to perform their daily routines, while both groups continued to take their usual medication. To ensure that no participant undertook intense physical activity and should, therefore, be excluded from the analysis, a logbook was used to record the type of physical activity undertaken (domestic or recreational) and the approximate number of hours per week. The time elapsed between the first assessment and reevaluation was eight weeks for both groups.

### 2.2. Assessments

As discussed above, assessments were conducted twice, once at baseline and another at nine weeks following completion of the eight-week intervention. The following variables were assessed.

#### 2.2.1. Pain Catastrophizing

Pain catastrophizing was measured with the validated Spanish version of the Pain Catastrophizing Scale (PCS) for people with FM. This is a self-administered scale consisting of 13 items with a score ranging from 0 “Not at all” to 4 “All the time.” It presents three dimensions: (i) rumination, (ii) magnification; and (iii) helplessness. A total score is yielded (ranging from 0–52), whereby higher scores are representative of greater pain catastrophizing. The reliability of the scale is excellent (ICC = 0.94) [46].

#### 2.2.2. Anxiety

Anxiety was measured with the validated Spanish version of the Hospital Anxiety and Depression Scale (HADS) especially with the anxiety subscale. This subscale consists of seven items with a score ranging from 0 to 3. A total score of more than 10 points indicates anxiety. A score ranging from 8–10 represents a borderline case and a score of less than 8 points represents no significant anxiety [47]. It has shown an excellent reliability (ICC = 0.85) [48].

#### 2.2.3. Depression

Depression was evaluated by the validated Spanish version of the Beck Depression Inventory-Second Edition (BDI-II) [49], which is a widely used 21-item self-report inventory that has been proven to be highly accurate for measuring the severity of depression in patients with chronic pain [50,51] Each of the 21 items scores from 0 to 3 with a total score of 63 points. A score of 0–13 points means that there is minimal depression, 14 to 19 points means a mild depression, 20–28 points indicate a moderate depression, and 29 or more points indicate severe depression [49]. It has shown good reliability (ICC between 0.73 and 0.86) [52].

#### 2.2.4. Stress

The Perceived Stress Scale-10 (PSS-10), which was validated for the Spanish population and whose reliability has been proven to be excellent (ICC = 0.82), was used for the stress assessment. It is a self-report instrument with 10 items that evaluate the level of perceived stress during the last month with a 5-point response scale (0 = never, 1 = almost never, 2 = sometimes, 3 = fairly often, 4 = very often). Higher scores indicate a higher perceived stress [53].

#### 2.2.5. Perception of Pain

The perception of pain was measured using two approaches, which include pain acceptance and pressure pain threshold.

Pain acceptance was evaluated by the 15-item Spanish adapted version of the Chronic Pain Acceptance Questionnaire in patients with FM [54] (CPAQ-FM), which is a 15-item self-administered inventory measuring the acceptance of pain. The items are rated on a 7-point scale from 0 (never true) to 6 (always true). Higher scores indicate higher levels of acceptance. This tool has shown good internal consistency or reliability (Cronbach’s α: 0.78).

The pressure pain threshold (PPT) was assessed using an algometer (WAGNER Force Dial TM FDK 20/FDN 100 Series Push Pull Force Gage, Greenwich, CT, USA) at each of the 18 tender points used to diagnose FM [55]. First, the presence and location of the tender points was confirmed via palpation and pen-marked by an experienced physiotherapist. The pressure threshold was then measured by applying the algometer directly to the tender point with the axis of the shaft maintained at 90° relative to the examining surface. The area of the algometer tip was 1 cm^2^ and the pressure values were reported in kg/cm^2^. The subject was instructed to verbally inform when pain or discomfort was initially felt. The procedure used has excellent intra-observer reliability [56]. The average of the PPT measured was used for subsequent analyses [24].

#### 2.2.6. Quality of Life

Quality of life was assessed with the Spanish validated version of the Revised Fibromyalgia Impact Questionnaire (FIQR). This is a multidimensional self-administered questionnaire with 21 items divided into three domains: (i) physical function, (ii) overall impact, and (iii) severity of symptoms. Each item is evaluated on an 11-point numeric rating scale from 0 to 10, with 10 being the ‘worst.’ The summed score for physical function (range 0 to 90) is divided by 3, the summed score for overall impact (range 0 to 20) is not modified, and the summed score for symptoms (ranging from 0 to 100) is divided by 2. The total FIQR score is the sum of such three domain scores. It has an excellent reliability (ICC = 0.82) [57].

#### 2.2.7. Physical Conditioning

We assessed both the subjective and the objective physical conditioning. To assess the subjective physical conditioning, we evaluated the self-perceived functional capacity. The objective physical conditioning was determined by evaluating endurance and functional capacity, power, and velocity, as described below.

1. Self-perceived functional capacity was assessed based on the “Physical Function” subscale of the FIQR (FIQR-PF). This subscale consists of nine items assessing the self-perceived abilities to perform daily living activities (e.g., walk for 20 min, climb one flight of stairs…). The maximal score is 30. The higher scores point to a poorer perception of physical function. It has shown a good reliability (ICC = 0.73) [57].
2. Endurance and functional capacity were assessed by the six-minute walk test (6MWT). Participants walked down a 15-m long hallway for a total of six minutes. Any contra-indications were checked before the test started, so heart rate, oxygen level, and Borg Rate of Perceived fatigue were recorded besides the main variable, i.e., the walked distance. Patients were allowed to take as many standing rests as necessary, but the timer kept going. The instructions given to the patients were: “Walk to the turnaround point at each end. I am going to use this counter to keep track of the laps you complete. You may stand and rest, but you should walk as fast as you are able. Remember that the aim is to walk as far as possible, but do not run.” This test has shown an excellent reliability (ICC = 0.91) [58].
3. Power was evaluated by the five-repetition sit-to-stand test (5STST) consisting of sitting down and standing up from an armless chair (43 cm high) five times as quickly as possible. Participants with arms crossed over their chest were instructed to stand up completely and make firm contact when sitting. Timing began at the command “ready-steady-go” and stopped when they sat after the fifth stand-up [59]. This test has shown an excellent reliability in adult women (ICC = 0.92) [60].Velocity was assessed by the Four-Meter Gait Speed Test (4mGST). The 4mGST consisted of walking a distance of 4 m at the usual pace. This test in addition to assessing the walking speed allows us to estimate the risk of disability for a given individual [61]. Both the test-retest and the inter-rater reliability have been shown to be excellent (ICC = 0.89 − 0.99 and ICC = 0.97, respectively) [62].

### 2.3. Statistics

All statistical analyses were performed with SPSS v.24 (IBM SPSS, Inc., Chicago, IL, USA). Standard statistical methods were used to obtain the mean and standard deviation (SD). Inferential analyses of the data were performed using two-way mixed multivariate analysis of variance (MANOVA) with an inter-subject factor called “group” having two categories (PEG and CG), and a within-subject factor called “treatment” having two categories (T0 and T1). Post-hoc analysis was conducted using the Bonferroni correction provided by the statistics package used, and the effect size was calculated using Cohen’s d. We also compared age, weight, height, and level of pain between groups using a one-way ANOVA to ensure that the two groups were similar at baseline. The normality and homoscedasticity assumptions were checked by Shapiro-Wilk and Levene tests, respectively. Type I error was established as < 5% (*p* < 0.05).

## 3. Results

### 3.1. Participants

**Figure 1:**
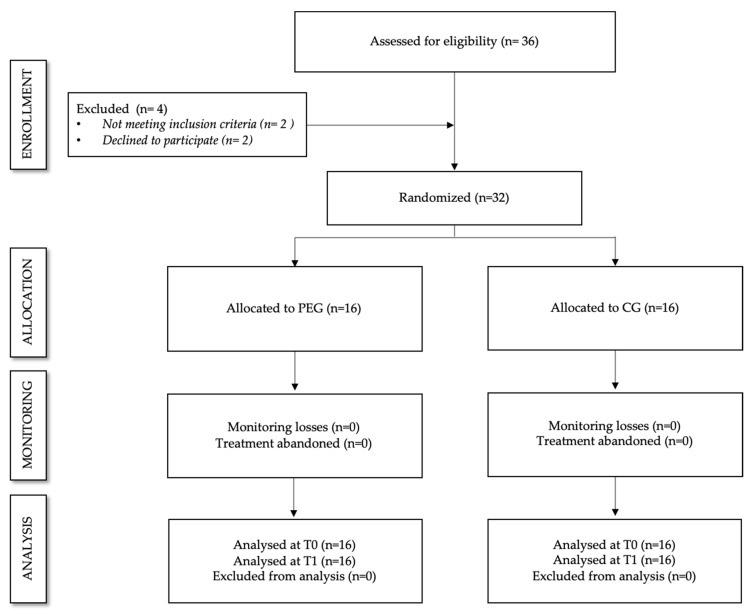
Flowchart according to CONSORT Statement for reporting randomized trials. PEG. Physical Exercise Group. CG. Control Group. T0. Pre-treatment assessment. T1. Post-treatment assessment.

Thirty-six subjects were assessed for eligibility. Two failed to meet inclusion criteria and two declined to participate. Therefore, 32 participants were included and then randomized (16 in PEG and 16 in CG) (Figure 1). The mean (SD) age for the participants was 53.06 (8.4) years for the PEG and 55.13 (7.35) years for the CG, weight, 70.35 (18.02) kg for the PEG, and 72.29 (13.94) kg for the CG, and height, 159.25 (6.2) cm for the PEG, and 160.38 (6.44) cm for the CG. There were no statistically significant differences in age, weight, height, and level of pain between groups (*p* > 0.05, data not shown). No incidents were reported at any point in time.

### 3.2. Intervenction Effects

The significant differences and the effect size among pre-treatment and post-treatment assessments (T0 and T1, respectively) for both groups and each variable are shown in Table 2 and Table 3 as well as the differences between groups for each assessed variable.

As shown in Table 2, all the psychological constructs assessed (i.e., pain catastrophizing, anxiety, stress, and depression) significantly improved in the physical exercise group (PEG) after the intervention with increases of 7.31, 1.87, 2.43, and 7.32 points, respectively. Statistically significant improvements were also observed in PEG for pain perception both in the pain acceptance with an increase of 4.94 points, and, in the average PPT, with a mean increase of 0.32 kg/cm^2^. Lastly, PEG also improved significantly by 9.98 points in quality of life. On the contrary, the CG failed to improve in any of the analyzed variables, and further exhibited a significantly poorer average PPT, with an average decrease of 0.25 kg/cm^2^.

In terms of the effect of the interventions on the individual’s physical conditioning, as noted in Table 3, participants belonging to PEG experienced a statistically significant improvement in their physical conditioning after the intervention. They improved their self-perceived functional capacity, as indicated by a 3.14-point increase in the FIQR-PF mean score. They also improved their endurance and functional capacity by increasing the average distance walked in the 6MWT test by 32 m. Furthermore, they improved their power and velocity, as observed by improved speed rates in both 5CRT and 4mGST of 6.85 and 0.49 s, respectively. Regarding the CG, no statistically significant differences were observed in any of the previously mentioned variables.

## 4. Discussion

This study shows that a low impact PE protocol combining endurance training (i.e., aerobic and resistance training aimed at improving endurance) and coordination is effective for improving psychological features (i.e., pain catastrophizing, anxiety, depression, stress), pain perception (i.e., pain acceptance and pressure pain threshold ), quality of life, and physical conditioning (i.e., self-perceived functional capacity, endurance and functional capacity, power, and velocity) in women with FM.

Pain catastrophizing refers to a set of exaggerated and ruminating negative cognitions and emotions during perceived or actual painful stimulation [2] and has been linked with adverse pain-related outcomes and FM-related disability [3]. PE has been posited as one of the most effective strategies to distract attention from pain [63] and reduce negative thoughts about pain, especially rumination [64]. In this regard, we observed a significant decrease in pain catastrophizing scores after the PE intervention. In line with these results, previous studies using PE alone or in combination with psychological/cognitive techniques, reported beneficial effects on pain catastrophizing in people with FM or chronic pain, as disclosed by a number of studies. This includes those conducted by Lazaridou et al. [35], in which a combined physical and psychological therapy (i.e., Yoga) was used, and those completed by Casey et al. [65] who applied PE combined with Acceptance and Commitment Therapy, or conducted by Seemts et al. [66] who combined aerobic exercise, mainly in water, with cognitive-behavioral treatment. These results suggest that psychological or/and physical techniques, either alone or in combination, may be beneficial to improve catastrophism in patients with chronic pain. However, the previously mentioned studies used standard PE programs without taking into account a potential aggravation of symptoms experienced by women with FM (i.e., fatigue), which has been posited as the main cause of low adherence to PE programs [38]. Our study reports that a customized low impact PE program, adapted to the individual’s self-perception of fatigue, is effective in improving pain catastrophizing. Conversely, no significant changes were observed in the CG.

This positive finding related to pain catastrophism was further confirmed by a significantly lower perceived pain, as indicated by higher pain acceptance and PPT values. Regarding pain acceptance, it has been associated with enhanced physical functioning in chronic pain patients. Likewise, the improved PPT may be due to a better physical conditioning [67,68], which, in turn, may lead to better pain acceptance [69]. Few authors have reported improvements in PPT after exercise programs [31,32] while using long-term interventions (i.e., 12–24 weeks), aquatic exercise, or psychological therapy. Therefore, their results are not entirely comparable. By contrast, CG subjects showed significantly poorer values for pain perception, as measured with an algometer, which may be due to the progressive physical deconditioning of these patients [67,68].

With regard to the other psychological variables analyzed (anxiety, depression, and stress), all of them significantly improved in the PEG. Improvements in anxiety may be due to the well-documented role of PE as a specific anxiety modulator [70]. In addition, anxiety has a direct relationship with pain acceptance [71], which, as discussed above, also improved in PEG. Some authors have documented the beneficial effects of PE on anxiety in people with FM [21,26,34]. The only study that analyzed the effect of a combined aerobic and resistance exercise protocol on anxiety reported a greater reduction than that obtained in our study (i.e., 41% compared to our 15%), which may be due to the well-known relaxing effects of warm water [34]. With regard to depression, we found positive results following the PE intervention with a similar [29,34] or even higher [20] reduction than that obtained in previous studies using combined aerobic and resistance PE protocols. This may be due to the release of neurotrophins triggered by PE, such as the brain-derived neurotrophic factor, as people with depression tend to display lower levels of this biomarker than their healthy counterparts, while PE induces its increase [72]. Lastly, the lowered stress levels observed in the current study suggests that PE could be a helpful approach to coping with stress, while also promoting stress resistance in women [64]. Previous studies have also concluded that moderate aerobic exercise [73] can reduce stress levels in people with FM, especially when working out in group settings, due to social interaction [74]. By contrast, we observed no improvements in the CG in any of the analyzed psychological variables. Overall, these results suggest that a combined low-intensity PE program, adapted to the individual’s symptoms, is effective in relieving anxiety, depression, and stress in women with FM.

As noted above, quality of life is impaired in people with FM [15]. Our PE protocol induced improvements in all the analyzed psychological constructs as well as in pain perception, which may have contributed to improving quality of life [75]. Many studies have shown that PE improves quality of life in the FM population, either through aerobic [20,23], resistance [19,26,37], and flexibility [24,26] exercises, protocols combining aerobic and resistance training [20], and specific modalities such as Tai-Chi [23]. However, such authors failed to include coordination exercises, which have been shown to challenge the sensory, cognitive, and musculoskeletal systems, and, thus, improve quality of life in older adults [76]. Yet, it has never before been implemented in women with FM. Thus, our results suggest that our PE protocol may be a useful tool to improve quality of life in women with fibromyalgia. In this regard, it would be interesting to apply the proposed exercise protocol on an ongoing basis, as it has been shown that long-term physical exercise positively affects quality of life in people with FM [77].

All variables related to subjective (i.e., self-perceived functional capacity) and objective (i.e., endurance and functional capacity, power, and velocity) physical conditioning improved significantly in the PEG, but not in the CG. This is of importance since both subjective and objective physical functions have been shown to be markedly impaired in women with FM, the former to a greater extent than the latter [14]. Our positive results on the subjective physical conditioning are noteworthy, since people with fibromyalgia who feel that they are unable to perform daily physical activities may avoid performing such activities and participating in therapeutic PE programs, which, in turn, may lead to objective physical deconditioning [14]. We, thus, evaluated objective physical conditioning by means of 6MWT, which is an inexpensive, relatively quick, safe, and a well-tolerated technique for the prediction of VO_2_ max [78], and may be considered an indirect measure of cardiorespiratory or maximal aerobic power fitness in this population. Furthermore, 5STST was chosen because not only lower limb strength and power are required, but also good coordination and balance are required. Therefore, it covers several important components of physical function [59,60]. Lastly, we assessed the 4mGST, since low gait speed has shown to be one of the main factors contributing to sarcopenia and, ultimately, to frailty [79]. Although the latter two variables have been mainly studied in older adults, they were used in the present study because women with FM have been show to display early aging and lower physical abilities compared to their age-matched healthy counterparts, which resembles healthy senior adults [80]. Our improvements in objective physical conditioning are in line with those reported by several authors following the implementation of different types of exercises, such as aerobic [22] or resistance exercises [22,25], or combined training (aerobic, resistance, flexibility, and patient education) [30].

Lastly, as pointed out before, lack of adherence seems to be typical in FM patients, which could be due to post-exercise soreness. The average adherence in reference studies was 85%, whereas adherence in our study was 100%. This may be due to the customized protocol we applied, which was duly tailored to each patient’s symptoms. The authors of the present study strongly believe that therapies aimed at FM patients should encourage participation by focusing on protocols with individualized work-loads, rather than relying on standard protocols.

### Limitations

The main limitation of the current study may be the small sample size. However, an *a priori* power analysis indicated that our sample size was sufficient. Future studies should confirm our findings in a larger population. However, therapeutic PE interventions should always be implemented in small groups in order to ensure proper performance of exercises, compliance with the protocol and, where necessary, an individualized correction of errors. Another limitation may be the fact that women were recruited from Fibromyalgia Associations, and, therefore, may present a different behavior than other FM patients. Regarding the protocol, a longer exercise program might have led to better results (i.e., differences between groups), and we did not perform any follow-up measurements to verify if the PE-induced benefits lasted in time. Lastly, since most FM patients are women, the current study was performed on women only, so this may bias the findings, which cannot be extrapolated to the general population.

## 5. Conclusions

The results obtained from this study show that a combined low-intensity PE program, including endurance training and coordination, improves pain catastrophizing in women with FM. Furthermore, the proposed protocol improves other psychological variables (i.e., anxiety, depression, and stress), perceived pain, quality of life, and physical conditioning in women with FM.

## Figures and Tables

**Table 1:** 10-exercise circuit included in the physical exercise group protocol.

1. Preacher curl while standing, palms facing forward
2. Leg extension while seated by lifting a sandbell
3. Bilateral dumbbell front raise while standing
4. Standing hip abduction with a soft elastic band
5. Chest lateral pull-ups while standing
6. Dumbbell shoulder external and internal rotation while standing
7. Sitting down and standing up from a chair without using arms
8. Throwing a ball above the head and catching it
9. Standing calf raise
10. Low Step-ups

**Table 2:** Effect of the intervention on the psychological constructs, perception of pain, and quality of life.

		Physical Exercise Group			Control Group	
	Pre-Treatment	Post-Treatment	Effect Size (d)	Pre-Treatment	Post-Treatment	Effect Size (d)
Pain catastrophizing	27.31 (11.55)	20.00 (10.86) *	0.65	28.25 (13.32)	27.06 (10.91)	-
Anxiety	11.81 (3.54)	9.94 (3.57) *	0.53	12.19 (4.07)	11.19 (3.69)	-
Depression	31.13 (9.06)	23.81 (7.93) *	0.86	29.31 (11.55)	27.94 (11.14)	-
Stress	25.31 (7.18)	22.88 (7.51) *	0.33	24.50(6.34)	24.75 (7.22)	-
Pain acceptance	38.00 (14.33)	42.94 (7.96) *	0.43	39.38 (14.67)	40.81 (13.54)	-
Pressure pain threshold (kg/cm^2^)	1.75 (0.98)	2.07 (1.03) *	0.32	1.76 (0.42)	1.50 (0.59) *	0.51
Quality of life	71.47 (14.21)	61.49 (17.65) *	0.62	62.44 (17.33)	67.07 (15.87)	-

Data are expressed as mean (SD), d: Cohen’s d effect size reported only when the differences between times were significant, *: *p* < 0.05.

**Table 3:** Effect of the intervention on participants’ physical conditioning.

		Physical Exercise Group			Control Group	
	Pre-Treatment	Post-Treatment	Effect Size (d)	Pre-Treatment	Post-Treatment	Effect Size (d)
Self-perceived functional capacity	20.06 (6.23)	17.46 (5.16) *	0.46	17.56 (7.28)	19.42 (6.03)	-
Endurance and functional capacity (m)	481.00 (71.23)	513.00 (64.84) *	0.47	493.19 (68.48)	497.31 (76.29)	-
Power (s)	18.18 (11.71)	11.33 (2.35) *	0.81	11.66 (3.06)	12.21 (3.01)	-
Velocity (s)	2.79 (0.39)	2.39 (0.27) *	1.19	2.47 (0.42)	2.36 (0.47)	-

Data are expressed as mean (SD), d: Cohen’s d effect size reported only when the differences between times were significant: *: *p* < 0.05.
